# Cephalometric Approach to the Occlusal Vertical Dimension Reestablishment

**DOI:** 10.1155/2014/920840

**Published:** 2014-07-03

**Authors:** João César Zielak, David Gulin Neto, Leonardo Fernandes da Cunha, Tatiana Miranda Deliberador, Allan Fernando Giovanini

**Affiliations:** Graduate Program in Dentistry, Positivo University, Rua Professor Pedro Viriato Parigot de Souza, 5300, 81280-330 Curitiba, PR, Brazil

## Abstract

The occlusal vertical dimension (OVD) refers to the length of the face as determined by the amount of separation of the jaws. Its determination is important for the manufacture of restorations. However, defining the correct occlusal vertical dimension for edentulous patients is one of the most important steps for function and esthetics rehabilitation. Cephalometry is a standardized method of assessing dental and facial proportions and their interrelation. Additionally, cephalometric analysis of the facial vertical dimension can establish an individual pattern for each patient. This analysis should become a permanent part of each patient's record. Hence, this study presented a case report with the use of cephalometry as an auxiliary tool in the rehabilitation of OVD. Clinical relevance showed that cephalometric analysis can be an accurate and convenient instrument to treatment planning and prognostic of oral rehabilitation. The reader should understand the clinical implications of using cephalometry as a tool in the rehabilitation of OVD.

## 1. Introduction

Determining the correct occlusal vertical dimension (OVD) for edentulous patients is one of the most important steps in making dentures with acceptable function and esthetics [1, 2]. Different techniques have been proposed to determine the correct measurement of the occlusal vertical dimension, such as the Sorenson Profile Scale and the measurement of vertical dimension from the base of the nasal septum to the inferior border of the chin [3, 4]. Some techniques have revealed dubious results [4, 5].

Willis suggested that when the teeth are in occlusion, the distance from the bony shelf under the nose to the bottom of the mandible should be coincident to the distance from the pupil of the eyes to the rima oris or parting line of the lips [5]. It was proposed that the occlusal vertical dimension should be measured, associated with the maximum biting force [6]. However, mandible controlling muscles can become tense when any type of mechanical recording device is placed in the mouth or on the head [7].

It is established that any alterations in the occlusal vertical dimension during clinical procedures may affect the stomatognathic system [8] and despite that, few practitioners have made attempts to use cephalometry as a diagnostic tool in prosthodontics; herein it was proposed that cephalometry could be an efficient method to help determine the OVD, since the cephalometry uses specific and predetermined point of bone references to obtain exact measurements [9, 10].

Hence, this study presented a case report using cephalometry as a tool in the rehabilitation of OVD.

## 2. Case Report

Two sisters, 64 (patient I) and 62 (patient II) years old, visited the Positivo University Dental Clinic, complaining of severe discomfort in relation to their upper and lower dentures (25 years of use), orofacial pain, chewing difficulty, headache, low self-esteem, and depression due to the aesthetic and functional facial impairments, affecting their quality of life and leading to physiological problems.

Both patients related the habit of squeezing and grinding teeth, which led to constant pain throughout the stomatognathic system. Clinical exams were conducted and demonstrated excessive wear of the base and teeth of upper and lower prostheses, leading to significant loss of occlusal vertical dimension and aging the patient's aesthetic profiles.

**Figure 1:**
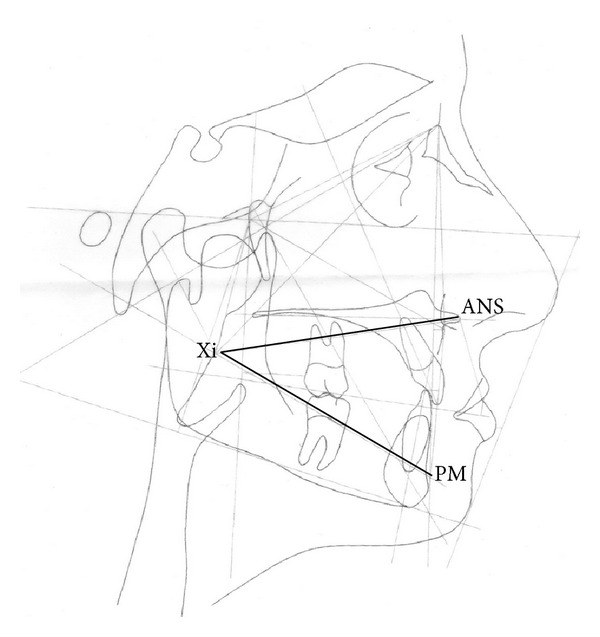
Lower face height (LFH) from the Ricketts lateral cephalometric analysis = angle between the planes formed by the anterior nasal spine to Xi point (ANS-Xi) and the Xi point to protuberance menti (Xi-PM). Coadjutant parameter to OVD reestablishment.

**Figure 2:**
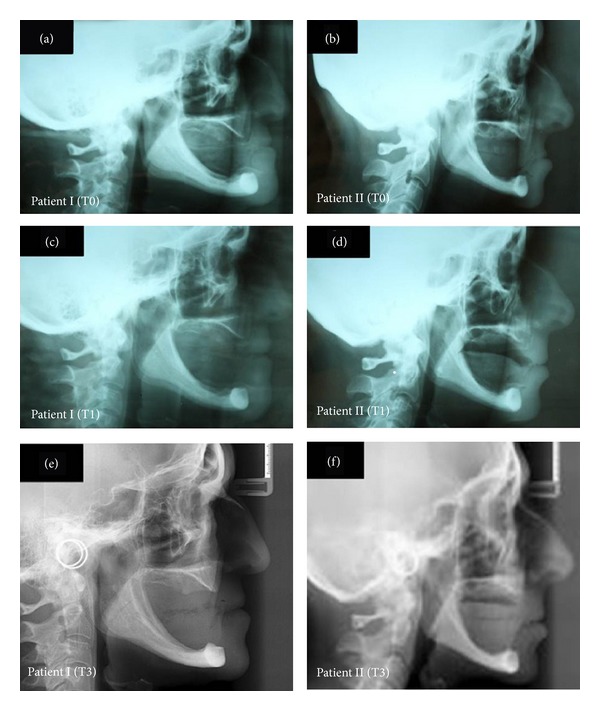
Lateral cephalometric radiograph of patients. (a) Patient I at baseline or T0. (b) Patient II at baseline or T0. (c) Patient I at trial insertion or T1. (d) Patient II at trial insertion or T1. (e) Patient I after four years of rehabilitation or T3. (f) Patient II after four years of rehabilitation or T3.

**Figure 3:**
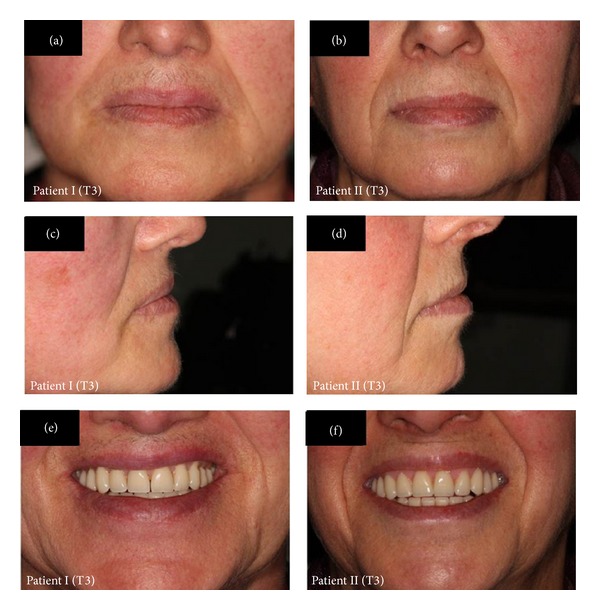
Images of clinical conditions of patients I and II after 4 years of rehabilitation or T3. (a) Resting posture of patient I. (b) Resting posture of patient II. (c) Facial profile of patient I. (d) Facial profile of patient II. (e) Smile of patient I. (f) Smile of patient II.

In order to reestablish function, a new pair of dentures was planned for each patient, using the lower face height (LFH) from the Ricketts lateral cephalometric analysis (Figure 1) as an auxiliary parameter for the OVD reestablishment. After the first clinical exams, a lateral cephalometric radiograph (LCR) was indicated (baseline, T0, Table 1; Figures 2(a) and 2(b)). The complete denture construction followed a conventional technique, such as primary and secondary impressions, occlusal recordings with rims, and OVD measurement with Willis compass, plus the trial insertion (triple-layer pressed polymethylmethacrylate teeth, Trilux, VIPI, Pirassununga, SP, Brazil). With the trial dentures in the mouth, the patients underwent a second LCR (T1, Table 1; Figures 2(c) and 2(d)). At this point, the LFH still showed a higher discrepancy of OVD, especially for patient I (35.38° compared to a minimum of 43°, with a clinical norm of 47.00 ± 4.00°; T1, Table 1). To increase the OVD in the lower and upper trial dentures, each angle degree was assumed as equivalent to 1 mm—naturally, in this step, the aesthetic aspect of the trial teeth dentures was also considered relevant. After the installation and necessary adjustments of the complete dentures, a third LCR was indicated (T2, Table 1). Only four years later, the patients were able to return, when another LCR was asked of both (T3, Table 1; Figures 2(e) and 2(f)). Declaring no discomfort symptoms or aesthetic problems, the patients decided not to renew the complete dentures at that time (Figure 3).

## 3. Discussion

Cephalometry is a standardized method of assessing dental and facial proportions and their interrelation [11]. In the technique, the patient is positioned within the cephalostat using adjustable ear rods placed within each auditory meatus. The mid sagittal plane of the patient is vertical and perpendicular to the X-ray beam, and the visual axis is horizontal so that the patient is positioned with the head held in the natural position [9]. Kundel states that image quality consists of three factors: image fidelity, image informativeness, and image attractiveness. All of these factors should be evaluated when the image of a given system is being assessed. Image fidelity refers to an accurate reproducibility of the structures [12]. In this case report, while the patients assumed an upper and lower arches position determined by the occlusal relation, its measurement was solely based on images of well-defined skeletal details, although the use of soft tissue references can also be used to define the face length [13, 14].

An accurate evaluation of the facial vertical dimension is essential to a successful prosthetic treatment, but the dentist cannot indiscriminately increase or decrease the OVD beyond the patient's physiologic requirements. A cephalometric analysis can represent a solid parameter from which the OVD can be established [3, 15]. OVD is unique for every individual and should not be changed [16] and the measurement of anatomic landmarks of the face can be controversial [3]. The simple Willis compass was developed to give proportional distances in the assessment of OVD [12]. However, because of face asymmetries, the use of only a few clinical anatomical landmarks can be questionable [17]. More recently, different methods of 2D and 3D studies of the soft tissues of the face are suggested [18].

Thus, considering the fact that the lower face height parameter from the Ricketts lateral cephalometric analysis is not obtained from any teeth planes or position, which can vary due to loss of elements or wearing of occlusal surfaces, it can be used as an individual pattern for each patient coadjutant in the establishment of OVD. This analysis can become a permanent part of each patient's record. It is unique for every patient and should not significantly vary throughout the lifetime [3].

## 4. Conclusion

A well-performed cephalometry can be an accurate and convenient tool to make the treatment planning and prognostic of the occlusal vertical dimension reestablishment more predictable.

## Figures and Tables

**Table 1:** Cephalometric data. Lower face height from the Ricketts lateral cephalometric analysis (LFH), at baseline (T0), trial insertion (T1), after installation and final adjustments of complete dentures (T2), and four years later (T3).

Date	Patient	LFH (°)	Clinical norm (°)
2007/06/25 (T0)	I	31.50	
	II	38.12	

2007/08/01 (T1)	I	35.38	47.00 ± 4.00
	II	39.97	

2007/08/16 (T2)	I	39.39	
	II	40.77	

2011/09/02 (T3)	I	43.83	45.00 ± 3.00
	II	46.94	
